# *N*-(4-Chloro­phen­yl)-4-methyl­piperidine-1-carboxamide

**DOI:** 10.1107/S1600536811024123

**Published:** 2011-06-25

**Authors:** Yu-Feng Li

**Affiliations:** a Microscale Science Institute, Department of Chemistry and Chemical Engineering, Weifang University, Weifang 261061, People’s Republic of China

## Abstract

In the title compound, C_13_H_17_ClN_2_O, the piperidine ring adopts a chair conformation and the N atom in that ring is close to pyramidal (bond angle sum = 357.5°). In the crystal, mol­ecules are linked into *C*(4) chains propagating in [010] by N—H⋯O hydrogen bonds.

## Related literature

For a related structure, see: Köhn *et al.* (2004 ▶).
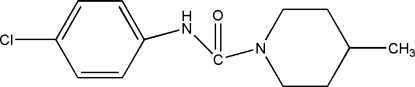


## Experimental

### 

#### Crystal data

C_13_H_17_ClN_2_O
*M* *_r_* = 252.74
Monoclinic, 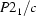

*a* = 13.286 (3) Å
*b* = 9.1468 (18) Å
*c* = 10.957 (2) Å
β = 95.36 (3)°
*V* = 1325.7 (5) Å^3^
*Z* = 4
Mo *K*α radiation
μ = 0.28 mm^−1^
*T* = 293 K
0.22 × 0.21 × 0.19 mm

#### Data collection

Bruker SMART CCD diffractometer
12608 measured reflections
3038 independent reflections
1999 reflections with *I* > 2σ(*I*)
*R*_int_ = 0.037

#### Refinement

*R*[*F* ^2^ > 2σ(*F* ^2^)] = 0.045
*wR*(*F* ^2^) = 0.154
*S* = 1.13
3038 reflections
154 parameters
H-atom parameters constrained
Δρ_max_ = 0.34 e Å^−3^
Δρ_min_ = −0.37 e Å^−3^

### 

Data collection: *SMART* (Bruker, 1997 ▶); cell refinement: *SAINT* (Bruker, 1997 ▶); data reduction: *SAINT*; program(s) used to solve structure: *SHELXS97* (Sheldrick, 2008 ▶); program(s) used to refine structure: *SHELXL97* (Sheldrick, 2008 ▶); molecular graphics: *SHELXTL* (Sheldrick, 2008 ▶); software used to prepare material for publication: *SHELXTL*.

## Supplementary Material

Crystal structure: contains datablock(s) global, I. DOI: 10.1107/S1600536811024123/hb5902sup1.cif

Structure factors: contains datablock(s) I. DOI: 10.1107/S1600536811024123/hb5902Isup2.hkl

Supplementary material file. DOI: 10.1107/S1600536811024123/hb5902Isup3.cml

Additional supplementary materials:  crystallographic information; 3D view; checkCIF report

## Figures and Tables

**Table 1:** Hydrogen-bond geometry (Å, °)

*D*—H⋯*A*	*D*—H	H⋯*A*	*D*⋯*A*	*D*—H⋯*A*
N2—H2*A*⋯O1^i^	0.86	2.33	2.940 (2)	128

Symmetry code: (i) 
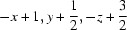
.

**Fig. 1.**
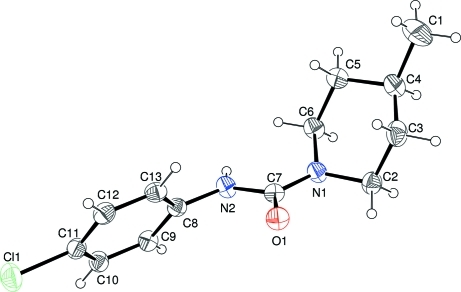
The structure of (I) showing 30% probability displacement ellipsoids.

## supplementary crystallographic information

### Comment

The crystal structure of the title compound, (I), is presented herein (Fig. 1). The six-membered rings (N1,C2, C3, C4, C5, C6) are in chair conformations. The molecules are linked into [010] chains by way of alternating N—H···O hydrogen bond linkages. The structure of a related compound has already been determined (Köhn *et al.*, 2004).

### Experimental

A mixture of 4-methylpiperidine (0.1 mol), and (4-chlorophenyl)carbamic chloride (0.1 mol) was stirred in refluxing ethanol (20 ml) for 4 h to afford the title compound (0.075 mol, yield 75%).  Colourless blocks of the title compound were obtained by recrystallization from ethanol at room temperature.

### Refinement

H atoms were fixed geometrically and allowed to ride on their attached atoms, with C—H distances = 0.93–0.97 Å; N—H = 0.86Å and with *U*_iso_(H) = 1.2*U*_eq_(C,*N*) or 1.5*U*_eq_(C_methyl_).

### Crystal data

**Table:**

C_13_H_17_ClN_2_O	*F*(000) = 536
*M**_r_* = 252.74	*D*_x_ = 1.266 Mg m^−^^3^
Monoclinic, *P*2_1_/*c*	Mo *K*α radiation, λ = 0.71073 Å
*a* = 13.286 (3) Å	Cell parameters from 1999 reflections
*b* = 9.1468 (18) Å	θ = 3.0–27.3°
*c* = 10.957 (2) Å	µ = 0.28 mm^−^^1^
β = 95.36 (3)°	*T* = 293 K
*V* = 1325.7 (5) Å^3^	Bar, colorless
*Z* = 4	0.22 × 0.21 × 0.19 mm

### Data collection

**Table:**

Bruker SMART CCD diffractometer	1999 reflections with *I* > 2σ(*I*)
Radiation source: fine-focus sealed tube	*R*_int_ = 0.037
graphite	θ_max_ = 27.5°, θ_min_ = 3.1°
φ and ω scans	*h* = −17→17
12608 measured reflections	*k* = −10→11
3038 independent reflections	*l* = −14→14

### Refinement

**Table:**

Refinement on *F*^2^	Primary atom site location: structure-invariant direct methods
Least-squares matrix: full	Secondary atom site location: difference Fourier map
*R*[*F*^2^ > 2σ(*F*^2^)] = 0.045	Hydrogen site location: inferred from neighbouring sites
*wR*(*F*^2^) = 0.154	H-atom parameters constrained
*S* = 1.13	*w* = 1/[σ^2^(*F*_o_^2^) + (0.0806*P*)^2^ + 0.0785*P*] where *P* = (*F*_o_^2^ + 2*F*_c_^2^)/3
3038 reflections	(Δ/σ)_max_ = 0.001
154 parameters	Δρ_max_ = 0.34 e Å^−^^3^
0 restraints	Δρ_min_ = −0.37 e Å^−^^3^

### Special details

**Table:**

Geometry. All esds (except the esd in the dihedral angle between two l.s. planes) are estimated using the full covariance matrix. The cell esds are taken into account individually in the estimation of esds in distances, angles and torsion angles; correlations between esds in cell parameters are only used when they are defined by crystal symmetry. An approximate (isotropic) treatment of cell esds is used for estimating esds involving l.s. planes.
Refinement. Refinement of F^2^ against ALL reflections. The weighted R-factor wR and goodness of fit S are based on F^2^, conventional R-factors R are based on F, with F set to zero for negative F^2^. The threshold expression of F^2^ > 2sigma(F^2^) is used only for calculating R-factors(gt) etc. and is not relevant to the choice of reflections for refinement. R-factors based on F^2^ are statistically about twice as large as those based on F, and R- factors based on ALL data will be even larger.

### Fractional atomic coordinates and isotropic or equivalent isotropic displacement parameters (Å^2^)

**Table:**

	*x*	*y*	*z*	*U*_iso_*/*U*_eq_	
Cl1	0.83830 (4)	0.11364 (7)	0.42550 (6)	0.0690 (2)	
O1	0.44390 (10)	0.14454 (13)	0.73592 (13)	0.0484 (4)	
C8	0.57283 (13)	0.30099 (18)	0.59955 (16)	0.0379 (4)	
N2	0.49079 (11)	0.36204 (15)	0.65583 (15)	0.0432 (4)	
H2A	0.4793	0.4543	0.6491	0.052*	
C12	0.63981 (15)	0.1297 (2)	0.46199 (18)	0.0452 (5)	
H12A	0.6304	0.0526	0.4067	0.054*	
C13	0.55876 (14)	0.1876 (2)	0.51610 (17)	0.0436 (4)	
H13A	0.4943	0.1500	0.4963	0.052*	
N1	0.35363 (11)	0.34976 (16)	0.77039 (16)	0.0447 (4)	
C9	0.66864 (14)	0.35849 (19)	0.62681 (18)	0.0421 (4)	
H9A	0.6783	0.4356	0.6820	0.051*	
C11	0.73497 (14)	0.1877 (2)	0.49098 (17)	0.0436 (4)	
C7	0.42888 (13)	0.27715 (18)	0.72121 (16)	0.0385 (4)	
C10	0.75036 (14)	0.3019 (2)	0.57244 (17)	0.0447 (4)	
H10A	0.8147	0.3406	0.5908	0.054*	
C6	0.30658 (13)	0.48409 (19)	0.71993 (19)	0.0473 (5)	
H6A	0.3479	0.5258	0.6603	0.057*	
H6B	0.3021	0.5549	0.7851	0.057*	
C2	0.29344 (15)	0.2735 (2)	0.85563 (19)	0.0485 (5)	
H2B	0.2864	0.3352	0.9264	0.058*	
H2C	0.3281	0.1847	0.8840	0.058*	
C4	0.13554 (16)	0.3713 (3)	0.7433 (2)	0.0639 (6)	
H4A	0.1244	0.4363	0.8119	0.077*	
C5	0.20188 (15)	0.4518 (3)	0.6593 (2)	0.0567 (5)	
H5A	0.2079	0.3931	0.5866	0.068*	
H5B	0.1694	0.5431	0.6335	0.068*	
C3	0.19038 (16)	0.2355 (3)	0.7956 (2)	0.0587 (6)	
H3A	0.1508	0.1902	0.8554	0.070*	
H3B	0.1971	0.1657	0.7302	0.070*	
C1	0.0326 (2)	0.3348 (5)	0.6765 (4)	0.1222 (14)	
H1A	0.0004	0.4231	0.6459	0.183*	
H1B	−0.0088	0.2881	0.7323	0.183*	
H1C	0.0415	0.2701	0.6094	0.183*	

### Atomic displacement parameters (Å^2^)

**Table:**

	*U*^11^	*U*^22^	*U*^33^	*U*^12^	*U*^13^	*U*^23^
Cl1	0.0609 (4)	0.0796 (5)	0.0696 (4)	0.0091 (3)	0.0222 (3)	−0.0124 (3)
O1	0.0568 (8)	0.0314 (6)	0.0577 (8)	0.0031 (6)	0.0080 (7)	0.0042 (6)
C8	0.0391 (9)	0.0341 (9)	0.0402 (9)	0.0000 (7)	0.0026 (7)	0.0040 (7)
N2	0.0428 (8)	0.0294 (7)	0.0585 (10)	0.0012 (6)	0.0104 (7)	0.0010 (7)
C12	0.0547 (11)	0.0415 (10)	0.0392 (10)	−0.0005 (8)	0.0029 (9)	−0.0049 (8)
C13	0.0443 (10)	0.0431 (10)	0.0418 (10)	−0.0047 (8)	−0.0034 (8)	−0.0020 (8)
N1	0.0400 (8)	0.0375 (8)	0.0575 (10)	0.0021 (6)	0.0089 (7)	0.0060 (7)
C9	0.0454 (10)	0.0371 (9)	0.0439 (10)	−0.0064 (8)	0.0044 (8)	−0.0028 (8)
C11	0.0473 (10)	0.0453 (10)	0.0389 (9)	0.0021 (8)	0.0082 (8)	0.0028 (8)
C7	0.0400 (9)	0.0340 (9)	0.0406 (9)	−0.0016 (7)	0.0002 (8)	−0.0005 (7)
C10	0.0406 (9)	0.0485 (11)	0.0453 (10)	−0.0060 (8)	0.0053 (8)	−0.0011 (8)
C6	0.0474 (10)	0.0351 (9)	0.0600 (12)	0.0049 (8)	0.0080 (9)	0.0012 (8)
C2	0.0516 (11)	0.0455 (10)	0.0496 (11)	−0.0007 (9)	0.0109 (9)	0.0035 (9)
C4	0.0430 (11)	0.0858 (17)	0.0633 (14)	−0.0028 (11)	0.0065 (10)	0.0013 (12)
C5	0.0482 (11)	0.0655 (13)	0.0561 (12)	0.0051 (10)	0.0030 (10)	0.0064 (11)
C3	0.0559 (12)	0.0610 (13)	0.0609 (13)	−0.0165 (10)	0.0141 (10)	0.0005 (10)
C1	0.0510 (15)	0.184 (4)	0.128 (3)	−0.027 (2)	−0.0114 (18)	0.031 (3)

### Geometric parameters (Å, °)

**Table:**

Cl1—C11	1.7441 (19)	C6—C5	1.514 (3)
O1—C7	1.237 (2)	C6—H6A	0.9700
C8—C9	1.384 (2)	C6—H6B	0.9700
C8—C13	1.384 (2)	C2—C3	1.503 (3)
C8—N2	1.416 (2)	C2—H2B	0.9700
N2—C7	1.379 (2)	C2—H2C	0.9700
N2—H2A	0.8600	C4—C5	1.522 (3)
C12—C11	1.381 (3)	C4—C3	1.524 (3)
C12—C13	1.382 (3)	C4—C1	1.526 (3)
C12—H12A	0.9300	C4—H4A	0.9800
C13—H13A	0.9300	C5—H5A	0.9700
N1—C7	1.353 (2)	C5—H5B	0.9700
N1—C2	1.462 (2)	C3—H3A	0.9700
N1—C6	1.463 (2)	C3—H3B	0.9700
C9—C10	1.386 (3)	C1—H1A	0.9600
C9—H9A	0.9300	C1—H1B	0.9600
C11—C10	1.377 (3)	C1—H1C	0.9600
C10—H10A	0.9300		
			
C9—C8—C13	119.49 (16)	H6A—C6—H6B	108.1
C9—C8—N2	119.08 (16)	N1—C2—C3	111.16 (17)
C13—C8—N2	121.42 (16)	N1—C2—H2B	109.4
C7—N2—C8	121.65 (14)	C3—C2—H2B	109.4
C7—N2—H2A	119.2	N1—C2—H2C	109.4
C8—N2—H2A	119.2	C3—C2—H2C	109.4
C11—C12—C13	119.19 (17)	H2B—C2—H2C	108.0
C11—C12—H12A	120.4	C5—C4—C3	109.76 (18)
C13—C12—H12A	120.4	C5—C4—C1	111.1 (2)
C12—C13—C8	120.52 (17)	C3—C4—C1	112.2 (2)
C12—C13—H13A	119.7	C5—C4—H4A	107.9
C8—C13—H13A	119.7	C3—C4—H4A	107.9
C7—N1—C2	119.25 (15)	C1—C4—H4A	107.9
C7—N1—C6	124.54 (16)	C6—C5—C4	112.92 (18)
C2—N1—C6	113.70 (15)	C6—C5—H5A	109.0
C8—C9—C10	120.46 (17)	C4—C5—H5A	109.0
C8—C9—H9A	119.8	C6—C5—H5B	109.0
C10—C9—H9A	119.8	C4—C5—H5B	109.0
C10—C11—C12	121.20 (17)	H5A—C5—H5B	107.8
C10—C11—Cl1	119.10 (15)	C2—C3—C4	111.12 (19)
C12—C11—Cl1	119.69 (14)	C2—C3—H3A	109.4
O1—C7—N1	123.05 (16)	C4—C3—H3A	109.4
O1—C7—N2	121.51 (16)	C2—C3—H3B	109.4
N1—C7—N2	115.41 (15)	C4—C3—H3B	109.4
C11—C10—C9	119.13 (17)	H3A—C3—H3B	108.0
C11—C10—H10A	120.4	C4—C1—H1A	109.5
C9—C10—H10A	120.4	C4—C1—H1B	109.5
N1—C6—C5	110.16 (16)	H1A—C1—H1B	109.5
N1—C6—H6A	109.6	C4—C1—H1C	109.5
C5—C6—H6A	109.6	H1A—C1—H1C	109.5
N1—C6—H6B	109.6	H1B—C1—H1C	109.5
C5—C6—H6B	109.6		

### Hydrogen-bond geometry (Å, °)

**Table:**

*D*—H···*A*	*D*—H	H···*A*	*D*···*A*	*D*—H···*A*
N2—H2A···O1^i^	0.86	2.33	2.940 (2)	128

Symmetry codes: (i) −*x*+1, *y*+1/2, −*z*+3/2.
